# Age in addition to RETTS triage priority substantially improves 3-day mortality prediction in emergency department patients: a multi-center cohort study

**DOI:** 10.1186/s13049-023-01123-8

**Published:** 2023-10-18

**Authors:** G. Malmer, R. Åhlberg, P. Svensson, B. af Ugglas, E. Westerlund

**Affiliations:** 1 Karolinska Institutet Department of Clinical Sciences, Danderyd Hospital Division of Medicine, Stockholm, Sweden; 2 Department of Emergency Medicine, Karolinska University Hospital, Solna, Stockholm, Sweden; 3 Department of Clinical Science and Education, Södersjukhuset, Karolinska Institutet, Stockholm, Sweden; 4 Department of Medicine, Karolinska Institutet, Solna, Stockholm, Sweden

**Keywords:** RETTS, Primary complaint, Triage, Risk factors, Emergency Medical Service, Hospital, Emergency medicine, Age factors, Mortality, Predictive value of tests, Observational study

## Abstract

**Background:**

Previous studies have shown varying results on the validity of the rapid emergency triage and treatment system (RETTS), but have concluded that patient age is not adequately considered as a risk factor for short term mortality. Little is known about the RETTS system’s performance between different chief complaints and on short term mortality. We therefore aimed to evaluate how well a model including both RETTS triage priority and patient age (TP and age model) predicts 3-day mortality compared to a univariate RETTS triage priority model (TP model). Secondarily, we aimed to evaluate the TP model compared to a univariate age model (age model) and whether these three models’ predictive performance regarding 3-day mortality varies between patients with different chief complaints in an unsorted emergency department patient population.

**Methods:**

This study was a prospective historic observational cohort study, using logistic regression on a cohort of patients seeking emergency department care in Stockholm during 2012–2016. Patient visits were stratified into the 10 chief complaint categories (CCC) with the highest number of deceased patients within 3 days of arrival, and to “other chief complaints”. Patients with priority 1 were excluded.

**Results:**

The studied cohort contained 1,690,981 visits by 788,046 different individuals. The TP and age model predicted 3-day mortality significantly and substantially better than both univariate models in the total population and in each studied CCC. The age model predicted 3-day mortality significantly and substantially better than the TP model in the total population and for all but three CCCs and was not inferior in any CCC. There were substantial differences between the studied CCCs in the predictive ability of each of the three models.

**Conclusions:**

Adding patient age to the RETTS triage priority system significantly and substantially improves 3-day mortality prediction compared to RETTS priority alone. Age alone is a non-inferior predictor of 3-day mortality compared to RETTS priority. The impact on 3-day mortality prediction of adding patient age to RETTS priority varies between CCCs but is substantial for all CCCs and for the total population. Including age as a variable in future revisions of RETTS could substantially improve patient safety.

## Background

Triage is widely used in emergency care to allocate scarce medical resources to patients who would most likely suffer or be harmed by waiting for medical attention [1–3]. In addition to medical and surgical intervention, caring for the patient’s needs in emergency care includes such measures as caring, comforting and palliating [3]. The most widely employed and well-studied triage systems today are the Canadian Triage and Acuity Scale [4], Emergency Severity Index, Manchester Triage Scale, Australasian Triage Scale and South African Triage Scale [5, 6].

Rapid Emergency Triage and Treatment System (RETTS) is applied for triage in emergency departments (EDs) in 80% of all regions in Sweden [7] and it is also widely applied in Norway and to some extent in Denmark [7, 8]. Similar many other triage systems, it employs a 5-grade triage priority (TP) scale and is based on assessing vital signs including respiratory rate, oxygen saturation, heart frequency, systolic blood pressure, consciousness and body temperature, as well as a short, structured anamnesis by a trained nurse. The system assigns a TP of 1 for life threatening condition with need for immediate attention, ranging to 5 for no need or strongly limited need for emergency care. RETTS also assigns initial measures to be taken based on the evaluation, such as blood sampling and/or monitoring [7].

Even for the more widespread and well-studied triage systems mentioned above there is insufficient scientific support regarding their ability to predict short term mortality in the general unsorted population due to low study power or study populations limited to specific conditions. Studies assessing the predictive ability regarding triage performance over different age groups are rare, and the results are inconclusive [5, 9–13].

Previous studies have shown varying results on the predictive value of RETTS specifically, on short to medium term mortality. In a 2020 study on sepsis detection, RETTS did not perform better than random guessing of 3 day mortality (3dM) for patients with one or more deranged vital sign [14]. In a study on prehospital RETTS triage the same year with 4465 patients included, the sensitivity of RETTS for adults the area under the curve (AUC) for 48 h mortality prediction was 0.712 (99% CI 0.646–0.759) [15]. In a 2016 single center study on 96,512 patients, increasing age was associated with increased 1- and 30-day mortality also when adjusted for all vital signs included in the RETTS triage system [16] and in a 2019 study on 639,387 patient visits, it was demonstrated that the association between increased age and RETTS priority adjusted 7-day mortality was highest for low priority patients [17].

It is, however, unknown if the RETTS triage system’s ability to predict short term mortality varies between patients with different chief complaints. It is also unknown if adding age information to the triage system would impact its predictive ability, and whether any such impact is the same for different chief complaints.

With crowding and long times to physician assessment for low priority patients in EDs in Sweden and elsewhere [18–20], there is a need for emergency services to be able to better single out patients in need for urgent medical attention from patients with severe but less urgent conditions. Improving a triage system’s ability to identify patients at risk of dying within a short time could both reduce the risk of delayed intervention for patients with reversible conditions and reduce the suffering for patients with irreversible conditions.

All with regards to 3-day mortality, we therefore aimed to evaluate whether a model including both TP and age improved the prediction better than a univariate RETTS triage priority model, to compare the predictive performance of the univariate RETTS triage priority model to a univariate age model, and to evaluate whether these three models’ predictive performance varied between patients with different chief complaints. The aim was also to apply the above questions to a large unsorted ED patient population.

## Methods

This was an observational cohort study on a previously collected cohort of patients seeking ED care in 2012–2016, comprising approximately 1.7 million ED visits. Logistic regression was used to predict 3dM and Receiver Operating Characteristics (ROC) and its AUC were used to assess model performance.

### Setting

The study was carried out in the Stockholm region, with a both urban and rural population of between 2.1 and 2.2 million with a mixed ethnical composition. 27% of the inhabitants in the Stockholm region were persons born outside of Sweden. The region was served by seven different emergency hospitals with EDs, of which six utilized RETTS for triage. All EDs were open to anyone who might be in need of emergency hospital resources, whereas local urgent care centers and primary care clinics to some extent serve patients with acute but minor conditions or injuries, such as e.g. minor wounds or mild infections.

### Data sources and collection

The study was carried out on a previously created and validated database with a cohort containing all adult (≥ 18 years old) patient visits to any of the six general EDs in the urban and rural Stockholm region utilizing RETTS during 2012 to 2016 and who had a valid Swedish personal identity number. The database constitutes 1,880,509 separate visits by 876,527 different individuals. The clinical data in the database was originally acquired from hospital data storage systems. It was collected and validated prospectively as part of day-to-day clinical evaluation and treatment of patients in the ED and no additional measures on patients were done for the purpose of this research. Information on deaths was acquired from the nationwide Cause-of- Death Register, which contains information on all deaths occurring in Sweden [21]. The Swedish unique personal identity number (PIN) is issued to all Swedish legal residents and was used to link events and data between included data storages and registries [22]. An extensive validation and explanation of the utilized registries is presented by Laugesen et al. [23].

The original data contained adult patients with a PIN visiting general EDs. Patients without a valid PIN were excluded since patient PIN constituted the key for registry matching. It is not known how many patients were excluded due to invalid PIN, but the group consists only of short-term visitors or illegal residents and is assumed to be very small and not have a major impact on the results of this study.

There were 1,880,509 visits by patients above 18 years of age to the six EDs in the Stockholm region who used RETTS during 2012–2016. Patients who received TP 1 as first TP were excluded as they are typically not handled by the ED triage system, but rather arrive through prehospital care, are triaged before arrival, and are attended to immediately by physicians upon arrival. Patients who received TP 5 as first TP and were deceased on the same date or the date after arrival were excluded since TP 5 in the RETTS system constitutes patients who are either dead on arrival or for other reasons deemed not in need of emergency care. Patients with no TP assigned were also excluded from the study as they, similarly to TP 5 patients, to a large extent are patients who are directed away from the ED, who leave the ED before triage has been done, or who are dead on arrival. The remaining 1,690,981 patient visits (89.9%) by 788 046 different individuals were included. The number of patients lost to follow-up is unknown, but deemed very low, due to the Swedish cause of death register including all individuals deceased in Sweden and the very short follow up time.

### Analyses

Chief complaints were entered from a predefined list by a trained nurse at the front desk. All registered chief complaints were categorized, joining alternative spellings, synonymous meanings, and different symptoms of similar conditions into the same chief complaint category (CCC). For example, the chief complaints “Femur injury”, “Hip injury”, and “Hip/Thigh injury” were categorized into the CCC “Hip/Thigh/Femur injury”.

Patient age was calculated with granularity level of 1 day. Whether or not the patient was alive or dead within 3 days of arrival at the ED front desk was calculated, and the ten CCCs who had the largest number of deceased patients within 3 days of arrival at the ED were extracted to ten separate strata. All other patient visits were categorized into one stratum named “Other chief complaints”. In cases where patients’ TP was changed during the ED visit, patients’ first acquired triage priority was selected as TP.

Three logistic regression models were fitted to the data for each CCC, as well as for “Other chief complaints” and for the total population, with 3dM as outcome: One univariate model with the first TP assigned to the patient as predictor (TP model) and one univariate model with patient age as predictor (age model). The third model was bivariate with both first TP and patient age as predictors (TP and age model). Each model was then used to predict 3dM. The quantitative performance for each model was evaluated using ROC curve analysis with AUC as quantitative measure of model performance. For each CCC, two-sided significance tests of the AUC were performed between each possible pair of models using DeLong, with *p*-values computed with an unpaired t-test. Significance was evaluated on the 0.05 level and statistical analysis was carried out in R version 3.5.3, utilizing package “pROC” [24, 25].

The study was approved by the Regional Ethics Committee in Stockholm, with Dnr 2014_1822-31 and amendment Dnr 2020-01691.

## Results

**Fig. 1:**
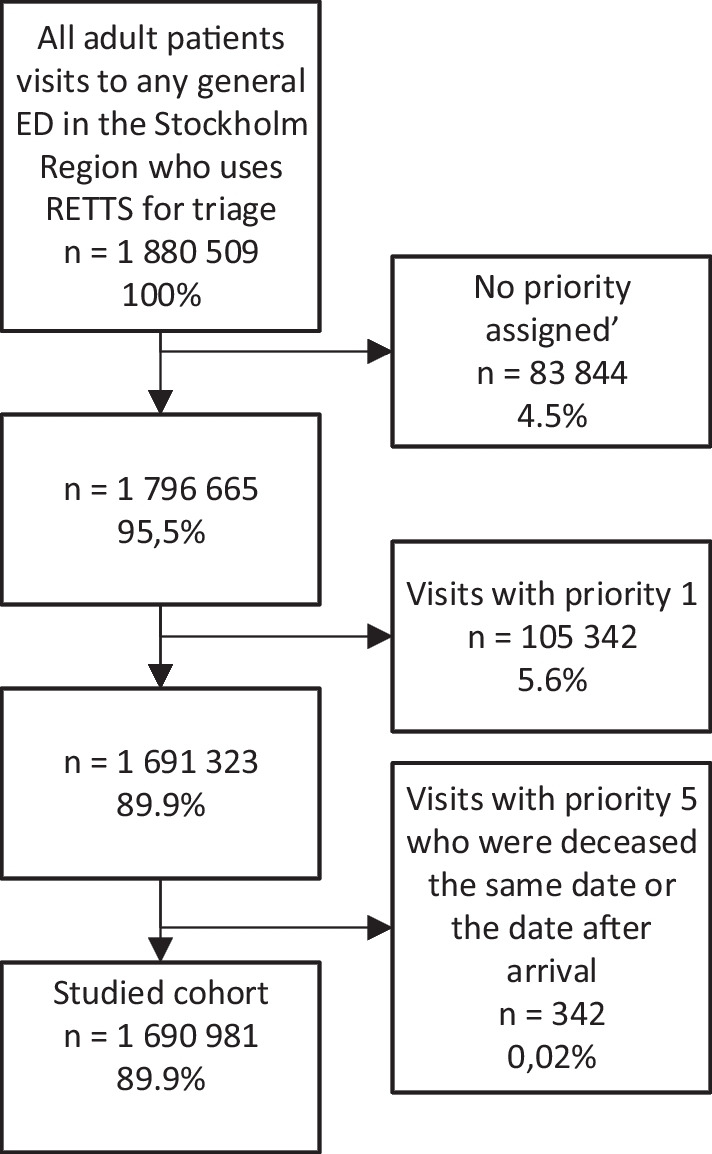
Inclusion and exclusion criteria, numbers, and percentages of population

The flowchart which resulted in a final study cohort of 1,690,981 ED-visits is presented in Fig. 1. Baseline characteristics of the study cohort are presented in Table 1. A total of 2854 patients died within 3 days of the ED-visit, which corresponded to a 3 dM of 0.17%. The ten specific CCCs analyzed included 47.5% of all patient visits and 67.1% of the patients who were deceased within 3 days. The categorizations are presented in Table 2 in “Appendix A”.

**Table 1:** Patient characteristics on arrival to the ED

Patient characteristics	Total visits		Alive after 3 days		Deceased after 3 days		3-day mortality (%)
	n	%	n	%	n	%	
Total	1,690,981	(100%)	1,688,127	(100%)	2854	(100%)	0.17
*Hospital*							
Danderyd	374,431	(22.1%)	373,845	(22.1%)	586	(20.5%)	0.16
Huddinge	316,450	(18.7%)	315,922	(18.7%)	528	(18.5%)	0.17
Norrtälje	103,556	(6.1%)	103,288	(6.1%)	268	(9.4%)	0.26
Solna	319,857	(18.9%)	319,428	(18.9%)	429	(15.0%)	0.13
Södertälje	130,216	(7.7%)	130,043	(7.7%)	173	(6.1%)	0.13
Södersjukhuset	446,471	(26.4%)	445,601	(26.4%)	870	(30.5%)	0.19
*Chief complaint category*							
Hip/Thigh/Femur injury	28,246	(1.7%)	28,058	(1.7%)	188	(6.6%)	0.67
Dyspnea	99,359	(5.9%)	98,769	(5.9%)	590	(20.7%)	0.59
Malaise/Fatigue	44,921	(2.7%)	44,666	(2.6%)	255	(8.9%)	0.57
Gastro-intestinal bleeding	15,297	(0.9%)	15,229	(0.9%)	68	(2.4%)	0.44
General infection or fever	48,235	(2.9%)	48,110	(2.8%)	125	(4.4%)	0.26
Neurological deficit	44,136	(2.6%)	44,063	(2.6%)	73	(2.6%)	0.17
Head injury	45,345	(2.7%)	45,271	(2.7%)	74	(2.6%)	0.16
Abdominal or flank pain	231,627	(13.7%)	231,293	(13.7%)	334	(11.7%)	0.14
Chest pain	139,786	(8.3%)	139,636	(8.3%)	150	(5.3%)	0.11
Painful or swollen extremity	106,558	(6.3%)	106,500	(6.3%)	58	(2.0%)	0.05
Others	887,471	(52.5%)	886,532	(52.5%)	939	(32.9%)	0.11
*Triage priority*							
2	246,987	(14.6%)	245,522	(14.5%)	1465	(51.3%)	0.59
3	747,558	(44.2%)	746,382	(44.2%)	1176	(41.2%)	0.16
4	512,103	(30.3%)	511,912	(30.3%)	191	(6.7%)	0.04
5	184,333	(10.9%)	184,311	(10.9%)	22	(0.8%)	0.01
*Age group*							
80 or older	239,679	(14.2%)	237,957	(14.1%)	1722	(60.3%)	0.72
60–79	476,694	(28.2%)	475,763	(28.2%)	931	(32.6%)	0.20
40–59	465,971	(27.6%)	465,798	(27.6%)	173	(6.1%)	0.04
18–39	508,637	(30.1%)	508,609	(30.1%)	28	(1.0%)	0.01
*Sex*							
Male	811,246	(48.0%)	809,762	(48.0%)	1484	(52.0%)	0.18
Female	879,735	(52.0%)	878,365	(52.0%)	1370	(48.0%)	0.16

Number of patients alive and deceased after 3 days (n) and 3-day mortality per exposure category. Percent of total population in each exposure category (%). Patient age is grouped in the table for readability, whereas patient age was entered in a 1-day granularity level in the statistical analysis

**Fig. 2:**
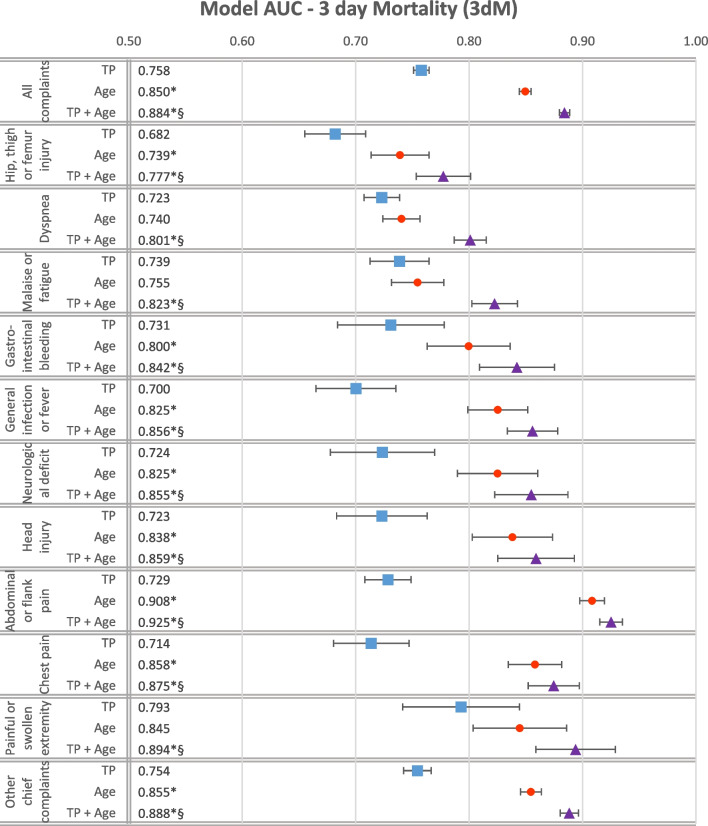
Model ROC AUC’s in predicting 3dM. Model ROC AUC’s in predicting 3dM, by CCC and with 95% confidence intervals drawn as whiskers. The assorted ED population is on top, individual CCCs are presented in order of decreasing 3dM, and the “Others” category is presented at the bottom. The numbers to the left indicate estimated AUC for each model. Asterisk (*) represents significantly larger AUC compared to the RETTS only model according to DeLong. Section mark (§) represents significantly larger AUC compared to the univariate age model according to DeLong. Significance was evaluated on the 0.05 level. The corresponding confidence intervals and *p*-values are presented numerically in “Appendix B”

**Fig. 3:**
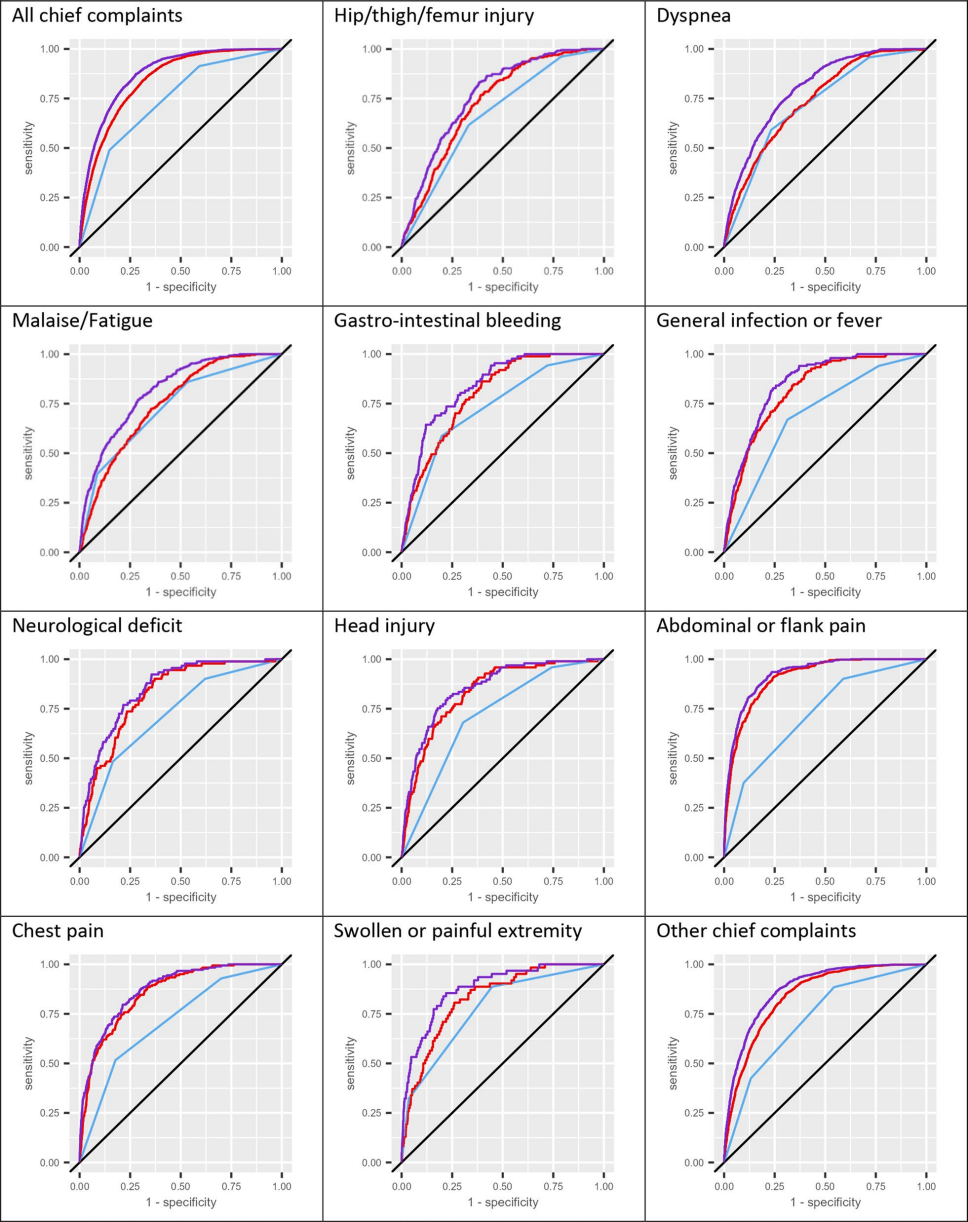
3 day mortality (3dM) prediction model ROC curves. The resulting ROC curves for each model by chief complaint category. The ROC curves represent the Univariate triage priority (TP) model (blue), the univariate age model (red) and the combined TP and age model (purple). The random classifier is represented by a diagonal black straight line. From left to right, top to bottom, the assorted ED population is on top, individual CCCs are presented in order of decreasing 3dM, and the “Others” category is presented at the bottom

The AUCs of the model ROC curves are represented in a forest plot in Fig. 2. The ROC curves of the individual models for each CCC are displayed in Fig. 3.

The combined TP and age model predicted 3dM significantly better than either of the univariate models in the total population as well as in each studied CCC. There were large differences in magnitude of improvement between the CCCs and between the contributions of the TP and age variables on model performance. Adding age information to the TP model generally made a larger contribution to model performance than adding TP to the age model.

The largest improvement in model performance by adding age information to the TP model was seen in patients presenting with “Abdominal or flank pain”, where the addition of age to the TP model increased AUC substantially from 0.723 (95% CI 0.708–0.749) to 0.925 (95% CI 0.915–0.935). For the univariate age model, the AUC for “Abdominal or flank pain” was 0.908 (95% CI 0.898–0.919). Similar patterns were observed for patients presenting with “Chest pain”, “Head Injury” or “General Infection or Fever”, whereas the “Dyspnea”, “Malaise/Fatigue” and “Painful or swollen extremity” CCCs displayed the smallest but still significant and substantial improvements in model performance from adding age information to the TP model.

The largest contributions in model performance from adding TP information to the age model were seen in the three CCCs “Hip, thigh or femur injury”, “Dyspnea” and “Malaise/Fatigue”. The smallest contributions were seen in the three CCCs “Chest pain”, “Abdominal or Flank Pain” and “Head Injury”, where no significant change in model performance could be observed from adding TP information to the univariate age model.

The univariate TP model performed best in the “Painful/swollen extremity” CCC, where the AUC was 0.793 (95% CI 0.741–0.845), and worst in the “Hip, thigh or femur injury” with an AUC estimate of 0.682 (95% CI 0.655–0.709). For the total population the AUC of the TP model was 0.758 (95% CI 0.751–0.765).

The best age model performance was seen in the “Abdominal or flank pain”, and the worst in the “Hip, thigh or femur injury” (AUC 0.739; 95% CI 0.655–0.709) CCC. For the total population the 3dM AUC estimate of the age model was 0.850 (95% CI 0.844–0.855).

The univariate age model had a significantly higher AUC than the TP model for the unsorted population and for each CCC except from patients presenting with “Dyspnea”, “Malaise/Fatigue” or “Painful/swollen extremity”. In these three CCCs there were no significant differences in AUC between the univariate models. The largest differences are notably seen in the “Abdominal or flank pain”, “Chest pain” and “General infection or fever” CCCs, which together constitute 24.9% of the studied population and 21.4% of the patients deceased after 3 days. The age model did not perform significantly worse than the TP model for any of the studied groups.

## Discussion

In this study in a large cohort of approximately 1.7 million unselected ED-visits, the addition of patient age information into a RETTS triage priority model significantly and substantially improved the model’s predictive performance for the total population and for each studied CCC. Further, a model based only on knowledge about the patient’s age was non-inferior to a model based on RETTS triage priority in predicting 3dM for the total population and each CCC. For all but three CCCs the age model was superior to the TP model in predicting 3dM. The study also showed that there were substantial differences between the CCCs in predictive improvement resulting from combining age and RETTS triage priority as well as between the CCCs in each model’s individual performance.

We chose 3dM as a proxy variable for acute illness in need of urgent emergency care for three reasons: First, one of the underlying purposes of this study is to evaluate RETTS’ validity in separating patients in dire need for urgent medical attention from patients with severe but less urgent conditions, and to which degree age could help in improving triage performance in this respect. Second, shorter mortality measures are at risk of suffering from patients surviving acute conditions for 1 or 2 days due to initial resuscitation efforts and intensive care, whereas longer mortality measures inevitably would include mortality from severe conditions that are relatively not non-urgent in the ED setting. The alternative of using in-hospital mortality would also suffer from abovementioned mortality from severe but non-urgent conditions, would be confounded by different follow-up times for different settings and conditions, and would suffer from loss to follow-up bias for patients who leave the hospital before dying from their condition. In contrast, mortality derived from the national cause of death register includes all deaths occurring nationwide during the follow-up period. We therefore believe that the 3dM measure is a balanced, reliable and relevant proxy variable for measuring conditions which are acute and severe in combination.

Clearly, there are conditions in the ED which are acute and require urgent attention due to insufferable pain or to avoid serious damage or disease but carry no or very little risk of death. It is important to bear in mind that the findings of this study do not address these conditions and not make the conclusion from age alone outperforming RETTS that age alone is a complete or superior substitute for a triage system. Also, there are patients deceased within 3 days whose course of disease would not have been altered by receiving a higher triage priority or more urgent attention. We believe, however, that according to the ethics of triage patients at risk of being deceased within 3 days should be identified and given attention swiftly, regardless of the reversibility of their condition [3]. Therefore, improving the triage system’s ability to identify patients with high 3dM has a value in and of itself.

The size and quality of the cohort in this study allows for extending previous research results to shorter term mortality and to specific CCCs. Compared to previous studies on smaller cohorts, our findings are in line with those of Ljunggren et al. 2016, showing 1- and 30-day mortality risk being higher for elderly people when adjusted for vital signs, gender, and co-morbidities [16], and with our previous study demonstrating an increased 30 day mortality risk associated with increasing age within each triage category [17]. Wireklint et al.’s [13] validation study on 74,845 patients in two EDs in southern Sweden estimated a ROC curve AUC of 0.735 for RETTS priority 1–4 and 0.873 for age in predicting 10-day mortality (10 dM). These values are in line with our findings regarding 3dM (0.758 and 0.850), but it is important to note that the study includes a different subset of triage priorities and and a longer mortality follow-up time.

### Strengths, validity and limitations of the study

The primary strength of this study is the large multicenter cohort, providing a first opportunity to evaluate 3dM with significant results on a subgroup level for unsorted ED patients. Also, the Swedish cause of death-registry provides for very few patients lost to follow-up, and thus high-quality outcome data both on hospitalized and non-hospitalized patients.

Triage priority, age and chief complaint is information typically provided early in the ED visit. Age and chief complaint may also easily be provided in a pre-hospital setting, why the results of this study regarding the age model to some extent may be valid for pre-hospital assessments such as e.g. ED front desk. This should, however, be applied with caution, since an inherent condition for all predictions in this study is that the patient has made the effort to make it to the ED.

The “Others” CCC includes complaints too fragmented with regards to chief complaint category to be analyzed for 3dM with the current data set, as the number of deceased in each CCC would be very small with a resulting loss of power. More data or a different study design could be used to analyze these chief complaints on a more granular level. Multiple specific studies have been done for single chief complaints within specific medical fields and we believe one of the strengths of this study to be the ability to compare age as a predictor between different CCCs. As a result, the”others” CCC is relatively large, contains very different conditions and has similar characteristics to the “all complaints” group. It can be noted, though, that even though the “others” CCC includes more than half the visits, it includes less than a third of the patients deceased within 3 days of their ED visit, which indicates that the study results include many of the more severe conditions in the specific CCCs. The standalone age results of this study could be applied to settings with similar health care systems, socioeconomic conditions, and demographics to that of the Stockholm region. The results on triage priority only apply directly to EDs utilizing the RETTS system. Notably, the results are only applicable to patients who are triaged to priority 2, 3, 4 or 5.

### Clinical applications

The simple logistic regression models used in this study makes for a transparent and easily computable method of assessing patient risk with the aid of computer- or application assistance, or a basis for further developing research based cutoff values for non-computer aided risk assessment in the ED or prehospitally [26]. Also, the simplicity of the included variables making for simplified application and introduction of the results in clinical practice, compared to e.g. frailty measures, additional testing or blood sampling. The results regarding the impact of age information for each of the ten CCCs which together account for more than two thirds of all patients which are deceased 3 days after an ED visit also provides for an opportunity to improve RETTS triage performance with more effectiveness than applying general age-based criteria on the overall ED patient group. This, in turn, could improve early identification of severely or acutely ill patients while preventing triage priority inflation and secondary excess mortality caused by ED crowding [20, 22, 27].

## Conclusion

Here we show that the addition of age to RETTS triage priority improves prediction of 3dM in a large and unsorted ED population comprising nearly 2 million visits, and for each of the ten deadliest CCCs. It is also shown that patient age alone is a better or non-inferior 3dM predictor than RETTS priority in all studied CCCs. The results of this study indicate that patient age could be used to alter priority of patients in order to increase patient safety and avoid unexpected adverse events in EDs utilizing RETTS for triage. In order to incorporate age into a triage algorithm, further methodological development and research would be required.

## Data Availability

The datasets used and/or analyzed during the current study are available from the corresponding author on reasonable request.

## Appendix A: Chief complaint categorizations

See Table 2.

**Table 2:** Chief complaint categorizations

Chief_complaint_Analysis_level	Chief_complaint_gr_lvl1	CHIEF_complaint_eng
Abdominal or flank pain	Abdominal pain	Abdominal pain
	Abdominal pain (medical)	Abdominal pain (medical)
	Flank pain	Flank pain
Chest pain	Chest pain	Chest pain
Dyspnea	Dyspnea	Dyspnea
		Dyspnea (surgical)
Extremity—swollen, pain	Extremity—swollen, pain	Extremity—swollen, pain
	Lower leg edema	Lower leg edema
General infection or fever	Fever	Fever
	Infection	Infection
Head injury	Head injury	Head injury
Hip/Thigh/Femur injury	Hip/Thigh/Femur injury	Femur injury
		Hip injury
		Hip/Thigh injury
Malaise/Fatigue	Malaise/Fatigue	Malaise
		Malaise/Fatigue
Neurological deficit	Absence attack	Absence attack
	Eye disorder	Eye disorder
	Facial palsy	Facial palsy
	Neurological deficit	Neurological deficit
	Numbness, prickling	Numbness, prickling
	Speech disorder	Speech disorder
	Stroke	Stroke
	Vision disorder	Vision disorder
	Weakness, paralysis	Weakness, paralysis
Gastro-intestinal bleeding	Blood in stool	Blood in stool
	Esophageal bleeding	Esophageal bleeding
	Gastro-intestinal bleeding	Gastro-intestinal bleeding
	Hematemesis	Hematemesis
	Melena	Melena
Others	–	–
	Abdominal injury	Abdominal injury
	Abortion complication	Abortion complication
	Abscess	Abscess
		Abscess (othopedic)
		Abscess (surgical)
	Acid or base lesion	Acid or base lesion
	Acute Stress or Crisis reaction	Acute Stress or Crisis reaction
		Crisis reaction
	Addison	Addison
	Allergic reaction	Allergic reaction
		Allergy
	Anemia	Anemia
	Ankle injury	Ankle injury
	Anxiety	Anxiety
	Apnea	Apnea
	Arrhythmia	Arrhythmia
	Ascites	Ascites
	Back injury	Back injury
	Back pain	Back pain
	Blister	Blister
	Blood disease	Blood disease
	Breast feeding failure	Breast feeding failure
	Bruise	Bruise
	Burn injury	Burn injury
	Cardiac arrest	Cardiac arrest
	Chemical exposure	Chemical exposure
	Childbirth	Childbirth
	Circulatory arrest	Circulatory arrest
	Cold, pulseless extremity	Cold, pulseless extremity
	Common cold	Common cold
	Confusion	Confusion
		Disturbed perception of reality
	Constipation	Constipation
	Cough	Cough
	Cramps	Cramps
	Decompression sickness	Decompression sickness
	Dementia	Dementia
	Deviating laboratory values	Deviating laboratory values
		Deviating laboratory values (medical)
	Deviating laboratory values (orthopedic)	Deviating laboratory values (orthopedic)
	Deviating laboratory values (surgical)	Deviating laboratory values (surgical)
	Diabetes	Diabetes
	Diarrhea	Diarrhea
	Direct admission	Direct admission
	Dizziness	Dizziness
	Drowning	Drowning
	Dysphagia	Dysphagia
	Dysphoria	Dysphoria
	Ear disorder	Ear disorder
	Ear injury	Ear injury
	Ear pain	Ear pain
	Eating disorder	Eating disorder
	Eczema	Eczema
	Elbow injury	Elbow injury
	Electrical injury	Electrical injury
	Examination requested by administrative authority	Examination requested by administrative authority
		Examination requested by administrative authority (medical)
		Examination requested by administrative authority (orthopedic)
		Examination requested by administrative authority (surgical)
	Eye injury	Eye injury
		Eye injury (medical)
		Eye injury (surgical)
	Facial edema/pain	Facial edema/pain
	Failure to thrive	Failure to thrive
	Foot injury	Foot injury
	Frost bite	Frost bite
	Genital injury	Genital injury
	Genital organ disorder	Genital organ disorder
	Genital organ disorder, female	Genital organ disorder, female
	Hand injury	Hand injury
	Hand/Arm injury	Hand/Arm injury
	Hanging	Hanging
	Headache	Headache
	Health checkup	Health checkup
	Hematuria	Hematuria
	Hemoptysis	Hemoptysis
	Hernia	Hernia
	Hyperglycemia	Hyperglycemia
	Hypertension	Hypertension
	Hypoglycemia	Hypoglycemia
	Hypotension	Hypotension
	Hypothermia	Hypothermia
	Immunodeficiency	Immunodeficiency
	Infection in lesion or skin	Infection in lesion or skin
		Infection in lesion or skin (ort)
		Infection in lesion or skin (surgical)
	Inhalation of gas or smoke	Inhalation of gas or smoke
	Insufficient/exaggerated food intake	Insufficient/exaggerated food intake
	Intoxication	Intoxication
	Itch	Itch
	Jaundice	Jaundice
		Jaundice (medical)
		Jaundice (surgical)
	Joint pain	Joint pain
		Joint pain (med)
	Knee/Lower leg injury	Knee/Lower leg injury
		Lower leg injury
	Laboratory results	Laboratory results
	Leg	Leg
	Lesion	Lesion
		Lesion (surgical)
	Lesion checkup	Lesion checkup
		Lesion checkup (surgical)
	Lesser complaint, not specified	Lesser complaint, not specified
		Lesser complaint, not specified (medical)
		Lesser complaint, not specified (orthopedic)
		Lesser complaint, not specified (surgical)
	Limping child	Limping child
	Localized infection	Localized infection
	Loss of consciousness	Loss of consciousness
	Loss of hearing	Loss of hearing
	Loud behavior	Loud behavior
	Lower arm injury	Lower arm injury
	Lowered consciousness	Lowered consciousness
	Lump	Lump
		Lump (medical)
		Lump (orthopedic)
		Lump (surgical)
	Mania/hypomania	Mania/hypomania
	Mastitis	Mastitis
	Menstruation disorder	Menstruation disorder
	Miscarriage complication	Miscarriage complication
	Missing	Missing
	Multiple trauma	Multiple trauma
	Nausea	Nausea
	Nausea/vomiting	Nausea/vomiting
	Neck injury	Neck injury
	Neck pain	Neck pain
	Non-specific psychiatric disorder	Non-specific psychiatric disorder
	Non-specific complaint	Non-specific complaint
	Nose bleed	Nose bleed
		Nose bleed (medical)
	Nose injury	Nose injury
	Object in airway	Object in airway
	Object in ear	Object in ear
	Object in esophagus	Object in esophagus
	Object in nose	Object in nose
	Object in rectum	Object in rectum
	Object in skin	Object in skin
		Object in skin (orthopedic)
		Object in skin (surgical)
	Object in vagina	Object in vagina
	Edema	Edema
	Organic psychiatric disorder	Organic psychiatric disorder
	Ostomy disorder	Ostomy disorder
	Penis disorder	Penis disorder
	Physical abuse	Physical abuse
	Physical abuse certificate	Physical abuse certificate
	Plaster disorder	Plaster disorder
	Post-partum complication	Post-partum complication
	Post-surgery complication	Post-surgery complication
	Pregnancy	Pregnancy
	Pregnancy disorder	Pregnancy disorder
	Prescription request	Prescription request
		Prescription request (medical)
		Prescription request (orthopedic)
		Prescription request (surgical)
	Probe or gastrostomy disorder	Probe or gastrostomy disorder
	Pseudo croup	Pseudo croup
	Psychiatric disorder	Psychiatric disorder
	Psychotic symptoms	Psychotic symptoms
	Rash	Exanthema
		Rash
	Respiratory tract infection	Respiratory tract infection
	Rib cage injury	Rib cage injury
	Scrotum disorder	Scrotum disorder
	Self-inflicted injury	Self-inflicted injury
	Serious event	Serious event (medical)
		Serious event (orthopedic)
		Serious event (surgical)
	Sexual abuse	Sexual abuse
	Shock	Shock
	Shoulder injury	Shoulder injury
	Sleeping disorder	Sleeping disorder
	Snake bite	Snake bite
	Social failure	Social failure
	Soft tissue injury	Soft tissue injury
		Soft tissue injury(orthopedic)
		Soft tissue injury(surgical)
	Sore throat	Sore throat
	Sting/Bite	Sting/Bite
		Sting/Bite from animal
	Strangulation	Strangulation
	Substance abuse	Substance abuse
	Suicide risk assessment	Suicide risk assessment
	Swallowed object	Swallowed object
	Symptoms from anus and rectum	Symptoms from anus and rectum
	Syncope	Syncope
	Thorax	Thorax
	Thorax/Back Injury	Thorax/Back Injury
	Toe, finger injury	Toe, finger injury
	Toe, finger pain	Toe, finger pain
	Tooth, mouth disorder	Tooth, mouth disorder
	Trauma	Trauma
	Traumatic amputation	Traumatic amputation
		Traumatic amputation (orthopedic)
	Tremor	Tremor
	Tumor	Tumor
	Twitching	Twitching
	Upper arm injury	Upper arm injury
	Urinary Catheter disorder	Urinary Catheter disorder
	Urinary tract disorder	Urinary tract disorder
	Urine retention	Urine retention
	Vaccination	Vaccination
	Vaginal bleeding	Vaginal bleeding
	Withdrawal	Withdrawal
	Worried parents	Worried parents
	Wrist injury	Wrist injury
	(blank)	(blank)

“chief_complaint_eng”: The stated complaint by the patient, categorized by the nurse according to hospital specific definitions, translated to English from Swedish

“chief_complaint_gr_lvl1”: “chief_complaint_eng” categorized into common chief complaints

“Chief_complaint_Analysis_level”: The Chief Complaint Categories (CCC) utilized for analysis in this study

## Appendix B: AUCs and confidence intervals for each model

See Table 3.

**Table 3:** AUC for each model in predicting 3 day mortality (3dM)

Chief complaint	Model	AUC estimate	AUC 95% C.I. low	AUC 95% C.I. high	*p*-value versus TP model	*p*-value versus age model
All complaints	TP	0.758	0.751	0.765	Reference	
	Age	0.850	0.844	0.855	< 0.001	Reference
	TP + Age	0.884	0.880	0.889	< 0.001	< 0.001
Hip/Thigh/Femur injury	TP	0.682	0.655	0.709	Reference	
	Age	0.739	0.714	0.765	0.003	Reference
	TP + Age	0.777	0.753	0.801	< 0.001	< 0.001
Dyspnea	TP	0.723	0.707	0.739	Reference	
	Age	0.740	0.724	0.757	**0.127**	Reference
	TP + Age	0.801	0.787	0.815	< 0.001	< 0.001
Malaise/Fatigue	TP	0.739	0.713	0.765	Reference	
	Age	0.755	0.732	0.778	**0.382**	Reference
	TP + Age	0.823	0.802	0.843	< 0.001	< 0.001
Gastro-intestinal bleeding	TP	0.731	0.684	0.778	Reference	
	Age	0.800	0.763	0.836	0.023	Reference
	TP + Age	0.842	0.809	0.875	< 0.001	< 0.001
General infection or fever	TP	0.700	0.665	0.735	Reference	
	Age	0.825	0.799	0.852	< 0.001	Reference
	TP + Age	0.856	0.834	0.878	< 0.001	< 0.001
Neurological deficit	TP	0.724	0.678	0.770	Reference	
	Age	0.825	0.790	0.861	< 0.001	Reference
	TP + Age	0.855	0.823	0.887	< 0.001	0.007
Head injury	TP	0.723	0.683	0.763	Reference	
	Age	0.838	0.803	0.874	< 0.001	Reference
	TP + Age	0.859	0.825	0.893	< 0.001	0.007
Abdominal or flank pain	TP	0.729	0.708	0.749	Reference	
	Age	0.908	0.898	0.919	< 0.001	Reference
	TP + Age	0.925	0.915	0.935	< 0.001	< 0.001
Chest pain	TP	0.714	0.681	0.747	Reference	
	Age	0.858	0.835	0.882	< 0.001	Reference
	TP + Age	0.875	0.852	0.897	< 0.001	0.002
Swollen or painful extremity	TP	0.793	0.741	0.845	Reference	
	Age	0.845	0.804	0.886	**0.105**	Reference
	TP + Age	0.894	0.859	0.929	< 0.001	< 0.001
Other chief complaints	TP	0.754	0.742	0.767	Reference	
	Age	0.855	0.845	0.864	< 0.001	Reference
	TP + Age	0.888	0.880	0.896	< 0.001	< 0.001

95% confidence intervals (C.I.) and the results of pairwise significance tests of all models’ areas under the receiver operating characteristic curves (AUC), within each chief complaint category (CCC). According to DeLong. Bold numbers indicate non-significant *p*-values. Significance was evaluated on the 0.05 level

## Abbreviations

3dM: 3-Day mortality
Age model: A univariate logistic regression model including patient age only
AUC: Area under the curve
CCC: Chief complaint category
C.I.: Confidence interval
ED: Emergency Department
PIN: Swedish personal identification number
RETTS: Rapid emergency triage and treatment system
ROC: Receiver operating characteristics
TP and age model: A bivariate logistic regression model including both RETTS triage priority and patient age
TP model: A univariate logistic regression model including RETTS triage priority only
TP: Triage priority
